# Dietary Supplementation of Sunflower Oil and *Lactiplantibacillus plantarum*-R11 Improves Meat Quality and Fatty Acid Composition in Crossbred (Boer × Saanen) Goats

**DOI:** 10.3390/ani16101540

**Published:** 2026-05-18

**Authors:** Lukman Abiola Oluodo, Patipan Hnokaew, Prayad Thirawong, Siriporn Umsook, Nursaadah Syahro Fitriyah, Chirawath Phatsara, Chompunut Lumsangkul, Napatsorn Montha, Saowaluck Yammuen-Art

**Affiliations:** 1 Department of Animal and Aquatic Science, Faculty of Agriculture, Chiang Mai University, Chiang Mai 50200, Thailand; 2 Graduate Program, Faculty of Agriculture, Chiang Mai University Under the CMU Presidential Scholarship, Chiang Mai 50200, Thailand; 3 Outreach Department, Rubber Research Institute of Nigeria, Benin City 1069, Nigeria; 4 Office of Research Administration, Chiang Mai University, Chiang Mai 50200, Thailand; 5 Department of Animal Science, Faculty of Agriculture at Kamphaeng Saen, Kasetsart University, Nakhon Pathom 73140, Thailand; 6 Department of Animal Science, College of Agriculture and Natural Resources, National Chung Hsing University, Taichung City 402202, Taiwan; 7 Center of Omics for High-Value Agriculture (AgOmics-CMU), Chiang Mai University, Chiang Mai 50200, Thailand

**Keywords:** goat, probiotics, *Lactiplantibacillus plantarum*, sunflower oil, fatty acid

## Abstract

This study investigated the individual and combined effects of SFO and LP supplementation on growth performance, carcass traits, meat quality, fatty acid profile, and serum biochemical indices in crossbred (Boer × Saanen) buck goats. Twenty-eight bucks (4 months old; 18.42 ± 2.03 kg) were randomly assigned to four treatments (*n* = 7): control, SFO (5–10 mL/day), LP (10 mL containing 10^7^ CFU/goat on alternate days), and SFO+LP. Growth performance was not affected; however, the feed conversion ratio (FCR) improved significantly (*p* < 0.001) in supplemented groups, with improved efficiency observed in SFO+LP, indicating enhanced feed utilization efficiency despite differences in feed intake. Carcass traits were unchanged. Meat quality improved in SFO and SFO+LP groups, as evidenced by lower pH at 45 min postmortem (*p* = 0.045) and reduced Warner–Bratzler shear force (*p* = 0.036), indicating enhanced tenderness. The combined SFO+LP treatment significantly increased C18:2 n6t (*p* = 0.036), total CLA (*p* = 0.003), and total PUFA (*p* = 0.001), indicating improved nutritional value of the lipids. Significant treatment × time interactions (*p* < 0.05) were observed for lipid and enzyme parameters, with values increasing over time in the supplemented groups, while glucose remained unchanged. These results indicate a synergistic interaction between SFO and LP, likely through modulation of rumen biohydrogenation and lipid metabolism, enhancing feed efficiency and meat functional quality without compromising metabolic health.

## Simple Summary

Improving the nutritional quality of goat meat while maintaining animal health is important in small ruminant production. This study evaluated the effects of sunflower oil (SFO) and *Lactiplantibacillus plantarum*-R11 (LP), used alone or in combination, on growth performance, meat quality, fatty acid composition, and blood biochemical parameters in crossbred (Boer × Saanen) buck goats. Growth performance and carcass traits were affected; however, feed efficiency improved in supplemented groups, particularly when SFO and LP were combined. Meat quality was enhanced, particularly with SFO alone or combined with the probiotic, as indicated by greater tenderness. The combined treatment also increased beneficial fatty acids, including total conjugated linoleic acid (CLA) and polyunsaturated fatty acids (PUFA). Blood analysis showed changes over time in some lipid-related parameters, but all values remained within normal physiological ranges, indicating no adverse effects on the animal’s health. These findings suggest that combining SFO with LP is a promising nutritional strategy to improve feed efficiency and enhance the nutritional value of goat meat.

## 1. Introduction

Goat production plays a significant role in supplying animal protein in many developing and tropical regions, where goats are valued for their adaptability, efficient feed utilization, and ability to thrive under diverse management systems [1]. Beyond productivity, attention has increasingly focused on improving meat quality and nutritional value to meet growing consumer demand for healthier animal products [2]. Dietary manipulation remains one of the most practical strategies for achieving these goals, particularly through lipid supplementation and rumen-modifying feed additives. Sunflower oil is a rich source of linoleic acid (C18:2 n-6). It has been widely used in ruminant diets to increase dietary energy density and to enhance the PUFA content of meat [3]. However, in ruminants, a large proportion of dietary unsaturated fatty acids undergo biohydrogenation in the rumen, which can limit their transfer to animal tissues [4,5].

Strategies that modulate rumen microbial activity may therefore enhance the efficiency with which dietary lipids are incorporated into muscle, improving both meat quality and nutritional composition. Probiotics, particularly lactic acid bacteria such as LP, have received increasing attention as natural feed additives that can improve rumen fermentation, nutrient utilization, and animal performance [6,7,8]. In addition to their effects on digestion, certain probiotic strains have been reported to influence lipid metabolism and fatty acid biohydrogenation, potentially enhancing the accumulation of health-promoting fatty acids such as CLA in ruminant products [9,10]. Nevertheless, responses to probiotic supplementation are often variable and may depend on diet composition and interactions with other feed components. *In vitro* studies have demonstrated that the combination of SFO and LP can modify rumen fermentation characteristics and biohydrogenation pathways [11], suggesting that dietary lipid supplementation coupled with probiotic-mediated rumen modulation may enhance the fatty acid profile of ruminant products. Consistent with this mechanistic evidence, Han et al. [12] reported that supplementation with a direct-fed microbial mixture containing *Lactobacillus acidophilus* and *Saccharomyces cerevisiae* of 5 g/animal/day containing *Lactobacillus acidophilus* (2.0 × 10^12^ colony-forming units/g; CFU) and *Saccharomyces cerevisiae* (5.0 × 10^11^ CFU/g) with 50 g/kg soybean oil in the diet increased the PUFA content of goat meat by 26%. These findings indicate a potential synergistic interaction between dietary oils and probiotics in improving the nutritional quality of meat.

Despite increasing interest in dietary lipid supplementation and probiotic use in ruminant production, findings remain inconsistent, particularly regarding their combined effects on growth performance, carcass traits, and fatty acid composition. While some studies suggest synergistic interactions between dietary oils and rumen-modulating microbes in enhancing beneficial fatty acids, other studies have reported inconsistent or limited responses depending on diet composition, microbial strains, and management conditions. These inconsistencies highlight the need for further investigation into the interactive effects of lipid sources and probiotics under controlled feeding conditions. Therefore, the objective of this study was to evaluate the individual and combined effects of SFO and LP supplementation on growth performance, carcass characteristics, meat quality, fatty acid profile, and serum biochemical indices in crossbred (Boer × Saanen) buck goats.

## 2. Materials and Methods

### 2.1. Production of Lactiplantibacillus plantarum-R11

The Chiang Mai University Institutional Biosafety Committee approved this protocol (CMUIBC0666001, Approval No. A0666001) before the commencement of the trial. *L. plantarum*-R11 was streaked onto de Man, Rogosa, and Sharpe (MRS) agar and incubated at 37 °C for 48 h, after which the isolate was stored at 4 °C. A single pure colony was revived in 10 mL of MRS broth and incubated for 24 h at 37 °C. The activated culture (1% *v*/*v*) was subsequently inoculated into fresh MRS broth in a sterile Erlenmeyer flask and incubated at 37 °C with agitation at 100 rpm for 15 h [13]. After incubation, the bacterial suspension was centrifuged using an Allegra X-22R Benchtop Centrifuge (Beckman Coulter, Indianapolis, IN, USA) at 11,700× *g* for 5 min. To minimize irritation during animal administration, the resulting bacterial pellet was washed twice with distilled water after centrifugation. The final pellet was stored at 4 °C before supplementation.

### 2.2. Animals, Diets, and Experimental Design

This research was conducted in adherence to international and national guidelines governing the care and use of research animals. The Animal Care and Use Committee at Chiang Mai University carefully evaluated and approved all experimental procedures (Protocol: RAGIACUC008/2566) before the commencement of the trial. The current study involved 28 crossbred (Boer × Saanen) buck goats from Mae Hia Farm, Chiang Mai University, Thailand, with an average age of 4 months and an average initial weight of 18.42 ± 2.03 kg. The feeding trial lasted for 104 days, including 14 days for adaptation to the diets and 90 days for data and sample collection. The goats were fed a basal diet consisting of whole corn plant (WCP) offered ad libitum and commercial concentrate (CO A NORTH Ltd., Lamphun, Thailand) provided at 1.0 kg/animal/day. The concentrate was offered in two equal portions (0.5 kg) at 7:00 and 16:00 h, respectively. Animals had free access to fresh drinking water and mineral block licks throughout the experiment. The chemical composition of WCP and commercial concentrate, as well as their fatty acid profiles, are presented in Table 1 and Table 2, respectively. The feed rations comprising each goat’s maintenance and production requirements [14]. The experiment used a completely randomized design (CRD), in which animals were randomly assigned to treatments. Initial body weight was considered to ensure balanced allocation; however, no significant differences in initial body weight were detected among treatments (*p* > 0.05; Table 3), indicating comparable baseline conditions. The twenty-eight goats were allocated to four groups (*n* = 7) based on body weight and housed individually. The groups comprised a control group (C_O_) receiving a basal diet without supplementation, alongside treatment groups receiving the basal diet supplemented with either SFO (T_SFO_), LP (T_LP_), or a combination of both (T_SFO+LP_) [15,16]. The experimental treatments differed only in the supplementation of SFO and LP. Sunflower oil was supplemented once daily, while LP was administered on alternate days. The SFO inclusion level was increased during the second phase of the experiment to account for the animals’ increased body weight. Additionally, the gradual increase allowed rumen microorganisms to adapt to dietary lipids, thereby minimizing potential negative effects on rumen fermentation, fiber digestion, and feed intake. This stepwise approach ensured rumen stability and efficient nutrient utilization throughout the experimental period. The study was designed to evaluate the functional effects of SFO as a linoleic acid (LA) source and of LP supplementation, rather than to compare isoenergetic or isonitrogenous diets. All treatment groups received the same basal diet, ensuring that observed differences could be attributed to the supplementation strategies.

The goats in the T_SFO_ and T_SFO+LP_ groups received daily oral supplementation with commercially available SFO (Naturel brand, Lam Soon Ltd.; Bangkok, Thailand) at a dosage of 5–10 mL. Initially, they were administered 5 mL per day for the first 45 days, followed by 10 mL per day for the subsequent 45 days. This supplementation was administered orally via a disposable syringe (NIPRO Corporation Ltd.; Ayutthaya, Thailand) in both periods. Additionally, goats in the T_LP_ and T_SFO+LP_ groups were orally inoculated with an anaerobic LP culture at a dose of 10mL containing 10^7^ CFU per goat. The LP culture was diluted with 10 mL of sterile water before administration. This oral supplementation was performed before morning feeding using a disposable syringe (NIPRO Corporation Ltd.; Ayutthaya, Thailand) on alternate days, for a total of 45 days from the start of the experiment to avoid excessive dose. The chemical composition of the experimental diet was determined using the proximate analysis method [17] and the detergent fiber analysis method [18]. Additionally, fatty acid profiles of the diet were determined using direct FAME synthesis [19].

Growth performance. The growth performance indices included the initial body weight (BW) at the commencement of the feed trial, and live body weight (LBW) was recorded every 14 days before offering the morning feeds. The goats were fed in the morning and afternoon at approximately 07:00 and 16:00 h, respectively. Daily rations were offered, and any feed refused was recorded. Feed conversion ratio was calculated as the ratio between dry matter intake (DMI, g/d) and weight gain (g/d). Average daily gain (ADG, g/d) was calculated as the difference between the final and initial BW divided by the number of days in the study. The experimental feeding period lasted 90 days, but growth parameters ended at 84 days, while carcass characteristics were determined after the feeding trial.

Carcass evaluation and sample collection. At the end of the experimental period, six goats were randomly selected from each group (out of seven animals per group, due to logistical constraints) on day 90 and subjected to slaughter following a 12 h fasting period to evaluate carcass traits. Animals were slaughtered humanely in accordance with standard animal welfare guidelines. Slaughter was performed following Halal procedures as practiced in Thailand. Briefly, animals were handled to minimize stress before slaughter, and a sharp knife was used to sever the carotid arteries, jugular veins, trachea, and esophagus to ensure rapid exsanguination. All procedures were carried out by trained personnel in accordance with hygienic and ethical standards. Animals were skinned, and abdominal and thoracic organs were detached and measured. The total weight of the digestive tract was recorded, and the empty body weight (EBW) was ascertained by deducting the alimentary tract’s content from the weight of the fasting live animal. The hot carcass weight and edible organs were promptly determined and expressed as a percentage relative to the fasted weight, providing an estimate of the dressing percentage. Subsequent steps involved removing and weighing non-carcass components comprising the head, pelt, feet, lungs, trachea, heart, liver, kidney, and testes. After slaughtering, the carcasses were halved and divided into prime cuts for further analysis (i.e., shoulder, leg, *longissimus dorsi* (LD) + tenderloin, rack, neck, brisket, flank, and legs). After 24 h at 4 °C, the carcass was stored at −20 °C to determine meat quality attributes and fatty acid composition in LD muscle.

Meat quality measurements. The pH of the LD muscles located between the 11th and 13th ribs was determined at 45 min and 24 h postmortem before sample collection, using a portable pH meter (Model 205, Testo, Lenzkirch, Germany). The collected LD muscles were prepared by slicing them into 2.5 cm thick slides from the anterior end. This measurement was carried out using a Konica Minolta Chroma Meter (Model CR-400, Minolta Camera Co., Ltd., Osaka, Japan) at 24 h postmortem, after 30 min of oxygenation of the 2.5 cm-thick LD muscles. The surface area of each muscle was measured six times at various angles after oxygenation to assess muscle color. The color components L*, a*, and b* were recorded. The proximate composition of the LD muscle was analyzed following AOAC [17] procedures: moisture by oven drying (Method 934.01), crude fat by Soxhlet extraction (Method 920.39), and crude protein by Kjeldahl method (Method 984.13).

Cooking loss measurement. The samples were thawed in a refrigerator at 4 °C for 20 h. For each treatment, approximately 80 g of LD muscle was excised and cut perpendicular to the direction of the muscle fibers, and the initial weight (W1) was recorded. The samples were then vacuum-sealed and cooked in a water bath at 75 °C (Techne Temperor, Staffordshire, UK) until the internal temperature reached 72 °C, as monitored with a thermometer (EBRO TTX 100, Ebro Electronic GmbH & Co. KG, Ingolstadt, Germany). After cooking, the samples were removed from the water bath and cooled under running water for 20 min until they reached room temperature. The meat surfaces were gently blotted with paper towels to remove excess moisture, and the samples were reweighed to obtain the final weight (W2) [20].

Cooking loss was calculated using the following formula:

$$\mathrm{Cooking}\;\mathrm{Loss}\;(\%)=\frac{W1-W2}{W1}\times 100$$

Warner-Bratzler shear force. The cooked samples were subsequently used to evaluate meat tenderness by measuring shear force, following the procedure described by Silva et al. [21]. The samples were cut into strips of 1.0 cm × 1.0 cm, and each strip was sheared perpendicular to the orientation of the muscle fibers using a Material Testing Machine (LR5K; Lloyd Instruments, West Sussex, UK). The instrument was fitted with a V-shaped blade and a 500 N load cell (S2M/500N, Force Transducer, HBM Singapore, Singapore) and operated at a constant crosshead speed of 60 mm/min. Each sample was measured eight times, and the maximum peak force (kg/f) was recorded and used as an indicator of meat tenderness.

Fatty acid analysis. Approximately 1.0 g of fresh minced LD muscles were methylated by direct fatty acid methyl esters (FAMEs) as described by O’Fallon et al. [19]. Before gas chromatography analysis, the collected hexane extract was concentrated under nitrogen and preserved at −20 °C. The composition of fatty acids was determined using a GC-7820A (Agilent Technologies Inc., Santa Clara, CA, USA) following the protocol by Anzhany et al. [22] and CP-Sil 88 fused-silica capillary column (100 m length × 0.25 mm diameter (i.d) and film thickness 0.20 μm; Agilent Technologies Inc., USA). The samples were classified by comparing their peak retention times with those of the FAME mixture standard (Food Industry FAME Mix, 30 mg/mL; RESTEK, Bellefonte, PA, USA), which provided isomeric profiles in the chromatogram. Detected fatty acids (FA) were classified into 9 groups (ratios and indexes): saturated fatty acids (SFA), monounsaturated fatty acids (MUFA), PUFA, CLA, Omega 3 (n3), Omega 6 (n6), and PUFA/SFA ratio [23]. Also, the indices of Δ9 desaturase enzyme activities (ID16 and ID18) in LD muscle were estimated according to the method described by Malau-Aduli et al. [24] as follows:

ID16 = 100(C16:1)/(C16:0 + C16:1)

ID18 = 100(C18:1)/(C18:0 + C18:1).

Serum Biochemical parameters. Blood samples were drawn through the jugular vein of each goat on days 1 and 90 of the trial; six goats were chosen randomly and without bias from each group as a representative. The same goats were bled 90 days into the trial. The samples were obtained using 4 mL vacutainers containing lithium heparin. After collection, whole blood samples were centrifuged using an Allegra X-22R Benchtop Centrifuge (Beckman Coulter, Brea, CA, USA) at 11,700× *g* for 5 min, followed by a second centrifugation at 3500× *g* for 15 min at 4 °C. The blood serum was transferred into 1.5 mL Eppendorf tubes and preserved at −20 °C until further analysis. The blood chemistry profile included glucose, triglyceride (TG), total cholesterol (TC), high-density lipoprotein (HDL), low-density lipoprotein (LDL), alanine transaminase (ALT), and aspartate transaminase (AST). These analyses were performed using an automated clinical chemistry analyser (BX-3010, Sysmex Asia Pacific Pte Ltd., Singapore).

## 3. Statistical Analysis

The study used a completely randomized design (CRD) with four treatments. Data on growth performance, carcass traits, meat quality, and fatty acid composition were analyzed using a general linear model in IBM SPSS Statistics (Version 26.0; 2019; IBM Corp., Armonk, NY, USA) as a completely randomized design. The statistical model used was:

$$Y_{ij}=\mu +T_{i}+\varepsilon_{ij}$$

where $Y_{ij}$ is the observed response, μ is the overall mean, $T_{i}$ is the fixed effect of dietary treatment, and is the residual error.

For serum biochemical parameters measured over time (Day 1 and Day 90), a repeated-measures ANOVA was applied, including the effects of treatment, time, and interaction. All data were tested for normality using the Shapiro–Wilk test before analysis. When significant differences were detected, means were separated using the Duncan multiple-range test. Results are presented as mean ± standard error of the mean (SEM), and statistical significance was declared at *p* < 0.05.

## 4. Results

Growth performance. Growth performance, as presented in Table 3, showed that initial and final body weight, average daily gain, and the concentrate-to-roughage ratio did not differ significantly (*p* > 0.05), whereas DMI and FCR were significantly affected by dietary treatments. Specifically, the control group exhibited the highest concentrate intake, roughage intake, and total DMI, while all supplemented groups, particularly T_LP_, showed significantly lower intake. Moreover, FCR showed highly significant differences (*p* < 0.05), with the control group having the poorest FCR (6.32) and the T_SFO+LP_ group showing the most efficient FCR (4.27), indicating that all supplemented diets improved FCR.

Carcass and Offal Characteristics. For carcass characteristics, as detailed in Table 4, no significant differences were observed for most parameters; several parameters showed trends (0.05 < *p* ≤ 0.01) among treatments. Finishing weight (*p* = 0.062), fast weight (*p* = 0.087), and slaughter weight (*p* = 0.057) tended to be higher in the T_SFO_ and T_SFO+LP_ groups than in the control. Similarly, hot carcass weight (*p* = 0.051) and cold carcass weight (*p* = 0.051). Among carcass cuts, trends were observed for shoulders (*p* = 0.095), LD + tenderloin (*p* = 0.084), and rack proportion (*p* = 0.081). Notably, the T_SFO+LP_ group had a higher proportion of LD + tenderloin than the other treatments. Regarding the percentage of offal cuts (relative to hot carcass weight), as presented in Table 5, no significant differences (*p* > 0.05) were revealed across all measured items, including head, pelt, feet, full digestive tract, heart, liver, kidney, lungs, spleen, and total offals.

Meat Quality of LD muscle. Table 6 reveals significant effects of dietary treatments on the meat quality of crossbred (Boer × Saanen) goats. The pH at 45 min showed a significant difference (*p* = 0.045), with the control group (C_O_) having a higher pH (6.51) compared to the significantly lower values (6.27–6.30) in groups supplemented with SFO (T_SFO_), LP (T_LP_), or both (T_SFO+LP_). No significant differences (*p* > 0.05) were observed in the color parameters (L*, a*, and b*) of the LD muscle among treatments. Shear force, an indicator of tenderness, also differed significantly (*p* = 0.036), with the C_O_ group exhibiting the highest force (4.54). At the same time, T_SFO_ (4.04) and, especially, T_SFO+LP_ (3.98) resulted in lower, more desirable values, with T_LP_ intermediate (4.24). Regarding chemical composition, moisture (*p* < 0.001) was significantly higher in the T_LP_ group (75.50) than in the others (72.70–73.20). Crude protein (*p* < 0.001) was significantly elevated in the T_SFO_ (22.43) and T_SFO+LP_ (23.12) groups, contrasting with the lower levels in C_O_ (20.97) and T_LP_ (20.44). Finally, crude fat (*p* < 0.001) was significantly lower in the T_LP_ group (1.66) than in the other treatments (2.93–3.00), which showed higher fat percentages.

### 4.1. Fatty Acid Profiles of LD Muscle

The fatty acid composition of the LD muscle, detailed in Table 7, displayed significant changes. While most individual saturated (SFA) and monounsaturated fatty acids (MUFA) and their totals were not significantly different (*p* > 0.05), the PUFA profile showed notable alterations. Specifically, C18:2 n6t (linoelaidic acid), total CLA, and total PUFA content were significantly different (*p* = 0.003, *p* = 0.028, *p* = 0.001). T_LP_ and T_SFO+LP_ treatments significantly increased total CLA and total PUFA compared to the control and T_SFO_ groups.

### 4.2. Serum Biochemical Indices

Serum biochemical indices were evaluated to assess the goats’ metabolic responses to dietary treatment over time, as detailed in Table 8. Significant treatment × time interactions (*p* < 0.05) were observed for triglycerides, total cholesterol, HDL, LDL, ALT, and AST, indicating that the changes in these parameters over time differed among treatment groups. Notably, goats receiving LP showed marked increases in TG, TC, HDL, LDL, ALT, and AST, indicating that changes in these parameters over time differed among treatment groups. Notably, goats receiving T_LP_ showed a marked increase in TG at day 90 compared to day 1. Similarly, HDL concentrations increased over time, particularly in the T_SFO+LP_ group compared to others. In contrast, glucose concentration was not significantly affected by treatment, time, or their interaction (*p* > 0.05).

## 5. Discussion

The absence of significant differences in final body weight and average daily gain among treatments indicates that dietary supplementation with SFO, LP, or their combination did not compromise growth performance in goats. In contrast, DMI and FCR were significantly affected by dietary treatments. The control group exhibited the highest concentrate intake, roughage intake, and total DMI, where all supplemented groups, particularly TLP, showed significantly lower intake. Estimated nutrient intake across treatments is presented in Supplementary Table S1, indicating that variations in crude protein and metabolizable energy intake were primarily associated with differences in voluntary feed intake under ad libitum feeding conditions. However, the marked improvement in FCR observed in goats receiving LP, and especially in those receiving the combined SFO+LP diet, suggests enhanced nutrient utilization efficiency. Improved feed efficiency without an associated increase in feed intake reflects favorable modulation of rumen microbial activity and nutrient digestibility, which has been widely associated with probiotic supplementation in ruminants [25,26]. In addition, dietary lipid supplementation has been shown to improve energetic efficiency by increasing dietary energy density and altering rumen fermentation patterns [27]. The lack of significant effects of dietary treatments on carcass traits and offal weights indicates that the observed improvements in feed efficiency did not translate into changes in carcass yield or tissue distribution. The absence of significant differences in carcass weight, dressing percentage, and most primal cuts among treatment groups aligns with previous findings on carcass characteristics and with studies showing that dietary supplementation with direct-feed microbial (DFM) or prebiotics did not alter carcass characteristics in goats [28,29,30]. However, the trend towards higher weight of LD + tenderloin in the T_SFO+LP_ group suggests that the combined supplementation may influence specific muscle deposition patterns, particularly in high-value cuts, in a longer feeding regimen.

Muscle pH is an important indicator of postmortem muscle metabolism, reflecting the rate of glycogen degradation and the subsequent accumulation of lactic acid after slaughter [31]. In the present study, the pH measured at 45 min postmortem (pH_45min_) represents the early rate of glycolysis in muscle. A relatively higher pH_45min_ generally indicates a slower rate of lactic acid production and delayed postmortem glycolysis. The results of this study suggest that dietary supplementation with SFO and LP did not accelerate early postmortem metabolic processes in goat muscle. Moreover, the ultimate pH measured at 24 h postmortem (pH_24h_) remained within the normal physiological range, indicating that the muscle glycogen reserves were sufficient to support an adequate postmortem decline in pH. These findings are consistent with the study of Liu et al. [32], who reported a decrease in pH_24h_ of the *longissimus thoracis* muscle in lambs following probiotic supplementation. However, Facciolongo et al. [33] reported an increase in pH_24h_ in rams supplemented with probiotics, suggesting that the effects of probiotic supplementation on postmortem muscle metabolism may vary across animal species, dietary composition, and experimental conditions. Overall, the results indicate that supplementation of SFO and LP did not adversely affect postmortem glycolysis [34]. The similarity in ultimate pH, color attributes, and cooking loss across treatments further confirms that dietary supplementation did not induce adverse effects on meat quality. However, Han et al. [12] reported that the effects of soybean oil and probiotic supplementation on meat color might be due to oil or probiotic dose, management, feed, or breed. The reduction in Warner–Bratzler shear force (WBSF) values observed in goats fed SFO and SFO+LP diets indicates enhanced meat tenderness following dietary lipid supplementation. Dietary fats can influence postmortem proteolysis, muscle fiber structure, and connective tissue characteristics, thereby contributing to improved tenderness [35]. In the present study, the combined SFO+LP treatment resulted in greater crude fat content in the LD muscle, reflecting increased intramuscular fat (IMF) deposition. Elevated IMF is known to improve tenderness through several mechanisms, including dilution of the connective tissue matrix, weakening of muscle fiber structural integrity, and the marbling-associated lubrication effect during mastication. Consistent with this, meat tenderness has been reported to be positively correlated with IMF content and negatively correlated with WBSF values [36,37,38]. Therefore, the higher fat content observed in the SFO+LP group likely contributed to the lower WBSF values recorded in this study. Collectively, these findings suggest a synergistic interaction between SFO and LP supplementation in enhancing meat tenderness, potentially through improved lipid incorporation and structural modification of muscle tissue. Differences observed in the proximate composition of the LD muscle further support the distinct metabolic roles of SFO and LP. The higher moisture content and lower crude fat concentration in the LP group indicate the production of leaner meat, which may result from improved nutrient partitioning toward muscle accretion rather than fat deposition, as previously reported for probiotic-supplemented ruminants [39]. Conversely, the increased crude protein and crude fat contents in goats fed SFO and SFO+LP diets reflect enhanced muscle protein deposition and lipid incorporation, consistent with the known effects of dietary fat supplementation on ruminant lipid metabolism [40].

Dietary supplementation with SFO markedly influenced the fatty acid composition of goat meat by increasing the concentration of linoelaidic acid (C18:2 n6t) and total PUFA. These changes are attributable to the high LA content of SFO and its partial escape from ruminal biohydrogenation [41]. The pronounced increase in CLA concentration in the SFO+LP group suggests that LP modulated rumen biohydrogenation pathways, thereby enhancing the conversion of LA to CLA. Similar synergistic effects between dietary lipids and rumen microbial activity on CLA synthesis have been reported previously [10,42]. This finding supports the hypothesis that combining lipid supplementation with probiotics can improve the nutritional quality of ruminant meat by enriching health-promoting fatty acids. In addition, an important component of our study was the evaluation of serum biochemical indices to assess the metabolic health and safety implications of dietary interventions. The significant treatment × time interactions observed for lipid-related parameters (TG, TC, HDL, and LDL) indicate that the metabolic response to supplementation evolved progressively throughout the experimental period. The increase in HDL concentration in the T_SFO+LP_ could facilitate reverse cholesterol transport and reflect improved lipid metabolism. This response may be associated with the enhanced deposition of PUFA and CLA observed in this study, as dietary unsaturated fatty acids are known to positively influence circulating lipoprotein profiles. Similar relationships between dietary lipids, fatty acid composition, and improved serum lipid metabolism have been reported in ruminants [43]. Modulating ruminal lipid metabolism, suggesting a synergistic effect of SFO and LP on meat quality and metabolic health. Furthermore, the observed changes in ALT and AST suggest metabolic adaptation rather than hepatic dysfunction, as values remained within physiological ranges [43]. This supports previous findings that direct-fed microbials can improve metabolic efficiency and reduce physiological stress without inducing adverse health effects [44].

## 6. Conclusions

Dietary supplementation with SFO and LP improved feed utilization efficiency in crossbred goats without affecting growth performance or carcass yield. Notably, although DMI was significantly lower in the T_SFO+LP_ group compared to the control, FCR was significantly improved, indicating enhanced feed efficiency due to the synergistic effect of combined supplementation. The combined supplementation of SFO and LP was particularly effective in enhancing meat tenderness and enriching the fatty acid profile of the LD muscle, notably by increasing PUFA and CLA concentrations. Importantly, the dietary interventions did not induce detrimental changes in serum biochemical indices, indicating that both individual and combined supplementation strategies are metabolically safe. Overall, the synergistic use of SFO and LP represents a practical nutritional approach for improving meat quality and nutritional value in goats while maintaining animal health and production efficiency. Nevertheless, further research is needed to elucidate the precise molecular mechanisms underlying SFO and LP’s effects on meat quality and fatty acid distribution, with a specific focus on their synergistic interactions to optimize future dietary strategies.

## Figures and Tables

**Table 1:** Dietary nutrient compositions of the whole corn plant and concentrate.

Item (DM Basis (g/kg))	Ingredient	
	WCP	Concentrate
Chemical composition		
Dry matter	220.00	888.30
Organic matter	880.00	846.70
Crude protein	86.00	130.40
Ether extract	22.40	22.90
NDF	746.30	604.00
ADF	464.90	438.80
ADL	34.90	48.40
Ca	4.70	25.60
Phosphorous	0.31	0.75

WCP: whole corn plant; DM: dry matter; NDF: neutral detergent fiber; ADF: acid detergent fiber; ADL: acid detergent lignin.

**Table 2:** Fatty acid composition of experimental diet and sunflower oil (g/100 g total fat).

Items ^1^	Experimental Diets		Linoleic Source
	Concentrate	Whole Corn Plant	Sunflower Oil
C12:0	3.48	1.19	ND
C14:0	3.2	2.71	0.11
C15:0	2.49	1.59	0.04
C16:0	17.47	19.82	6.76
C16:1	1.58	1.06	0.11
C17:0	1.44	1.53	0.07
C17:1	ND	9.78	ND
C18:0	7.33	ND	3.41
C18:1n9t	ND	3.41	ND
C18:1n9c	32.41	24.53	24.31
C18:2n6	24.28	17.83	63.85
C18:3n3	ND	16.55	ND
C21:0	6.32	ND	0.57
C22:0	ND	ND	0.77
ƩSFA	41.73	26.84	11.73
ƩMUFA	33.99	38.78	24.42
ƩPUFA	24.28	34.38	63.85

ND: Not detected; ^1^ C12:0: lauric; C14:0: myristic acid; C15:0: pentadecyclic acid; C16:0: palmitic acid; C16:1: palmitoleic acid; C17:0: margaric acid; C17:1: Heptadecenoic acid; C18:0: stearic acid; C18:1n9t: trans 9-octadecenoic acid; C18:1n9c: oleic cis-9; C18:2n6: linoleic acid; all trans-9,12; C18:3n3: α-linoleic acid; all-cis-9,12,15; C21:0: Heneicosylic acid; C22:0: Behenic acid.

**Table 3:** Growth performance of crossbred (Boer × Saanen) goats fed experimental diets.

Items ^1^	Dietary Treatments ^2^					
	C_O_	T_SFO_	T_LP_	T_SFO+LP_	SEM ^3^	*p*-Value
IBW (kg)	19.85	20.45	18.42	19.83	1.45	0.805
FBW (kg)	30.10	34.45	33.00	34.48	1.66	0.243
ADG (g/d)	139.08	166.67	156.54	174.41	14.77	0.441
DMI intake (g/d)						
Concentrate	628.51 ^A^	527.94 ^B^	500.10 ^B^	537.75 ^B^	27.04	0.020
Roughage	251.72 ^A^	240.14 ^A^	202.82 ^B^	208.10 ^B^	15.17	0.040
Total DMI	880.23 ^A^	768.08 ^B^	702.92 ^C^	745.85 ^B^	24.59	0.020
Concentrate: roughage	2.50	2.20	2.46	2.58	0.26	0.160
FCR	6.32 ^A^	4.61 ^B^	4.49 ^C^	4.27 ^D^	0.019	<0.001

^1^ IBW: initial body weight; FBW: final body weight; ADG: average daily weight; DMI: dry matter intake; FCR: feed conversion ratio; ^2^ C_O_: basal diet; T_SFO_: oral supplementation of sunflower oil + basal diet; T_LP_: oral supplementation of *L. plantarum*-R11 + basal diet; T_SFO+LP_: oral supplementation of sunflower oil + *L. plantarum*-R11 with the basal diet; ^3^ SEM: standard error of the mean (*n* = 7 goats per treatment); ^A,B,C,D^ mean values within a row with different superscript letters differ significantly (*p* < 0.05).

**Table 4:** Carcass characteristics of crossbred (Boer × Saanen) goats fed experimental diets.

Items	Dietary Treatments ^1^					
	C_O_	T_SFO_	T_LP_	T_SFO+LP_	SEM ^3^	*p*-Value
Finishing weight, kg	31.00	38.27	35.07	38.07	1.60	0.062
Fasting weight, kg	29.20	36.30	33.77	35.90	1.69	0.087
Slaughter weight, kg ^2^	28.23	35.40	32.70	35.07	1.56	0.057
Hot carcass weight, kg	12.80	16.77	15.93	16.73	0.87	0.051
Cold carcass weight, kg	12.54	16.43	15.61	16.4	0.86	0.051
Dressing yield %	44.05	46.14	47.14	46.60	1.27	0.433
Carcass Cuts, as % from hot carcass weight:						
Shoulder	16.93	15.97	15.39	15.36	0.35	0.095
Legs	4.54	5.59	5.12	5.24	0.25	0.113
LD + tender loin	7.61	7.07	7.87	8.74	0.37	0.084
Rack	5.45	6.93	6.04	5.42	0.36	0.081
Neck	14.79	17.43	15.94	15.89	1.43	0.684
Brisket	6.47	6.91	6.78	6.06	0.97	0.929
Flank	4.68	5.72	5.99	4.75	0.47	0.200
Prime cuts	91.42	93.60	90.25	87.53	2.00	0.312

^1^ C_O_: basal diet; T_SFO_: oral supplementation of sunflower oil + basal diet; T_LP_: oral supplementation of *L. plantarum*-R11 + basal diet; T_SFO+LP_: oral supplementation of sunflower oil + *L. plantarum*-R11 with the basal diet; ^2^ slaughter weight: after bleeding; ^3^ SEM: standard error of the mean (*n* = 6 goats per treatment).

**Table 5:** Percentage of offal cuts of slaughtered crossbred (Boer × Saanen) goats fed experimental diets.

Items	Dietary Treatments ^1^					
	C_O_	T_SFO_	T_LP_	T_SFO+LP_	SEM ^3^	*p*-Value
Offal Cuts, as % from hot carcass weight:						
Head	6.61	6.62	6.26	6.50	0.41	0.742
Pelt	9.13	8.94	8.63	9.40	0.55	0.828
Feet	2.91	2.64	2.75	2.73	0.10	0.438
Full digestive tract	26.93	25.61	26.02	26.38	1.22	0.888
Heart	0.40	0.40	0.37	0.41	0.01	0.495
Liver	1.40	1.34	1.47	1.37	0.07	0.835
Kidney	0.79	0.24	0.25	0.22	0.14	0.200
Lungs	11.69	15.69	14.35	14.66	0.84	0.425
Spleen	0.13	0.16	0.12	0.11	0.10	0.052
Testes	0.80	0.83	0.79	0.91	0.06	0.497
Total offals ^2^	4.62	4.08	4.10	4.10	0.28	0.718

^1^ C_O_: basal diet; T_SFO_: oral supplementation of sunflower oil + basal diet; T_LP_: oral supplementation of *L. plantarum*-R11 + basal diet; T_SFO+LP_: oral supplementation of sunflower oil + *L. plantarum*-R11 with the basal diet; ^2^ total offals (heart, liver, kidney, lungs, spleen and testes); ^3^ SEM: standard error of the mean (*n* = 6 goats per treatment).

**Table 6:** The meat quality of the LD muscle of crossbred (Boer × Saanen) goats fed experimental diets.

Items	Dietary Treatments ^1^					
	C_O_	T_SFO_	T_LP_	T_SFO+LP_	SEM ^2^	*p*-Value
pH value						
pH_45min_	6.51 ^A^	6.27 ^B^	6.23 ^B^	6.30 ^B^	0.05	0.045
pH_24h_	5.59	5.52	5.51	5.69	0.13	0.737
Meat color						
L* (lightness)	31.55	30.56	31.36	31.11	0.92	0.900
a* (redness)	6.19	6.25	6.69	6.78	0.19	0.238
b* (yellowness)	6.26	6.56	7.11	7.83	0.53	0.320
Cooking loss	23.67	24.05	27.49	24.89	2.46	0.727
Shear force (kg/f)	4.54 ^A^	4.04 ^AB^	4.24 ^C^	3.98 ^C^	0.10	0.036
Chemical composition, (%)						
Moisture	73.20 ^B^	73.00 ^B^	75.50 ^A^	72.70 ^B^	0.27	<0.001
Crude protein	20.97 ^B^	22.43 ^A^	20.44 ^B^	23.12 ^A^	0.43	<0.001
Crude fat	2.93 ^A^	3.00 ^A^	1.66 ^B^	2.94 ^A^	0.13	<0.001

^1^ C_O_: basal diet; T_SFO_: oral supplementation of sunflower oil + basal diet; T_LP_: oral supplementation of *L. plantarum*-R11 + basal diet; T_SFO+LP_: oral supplementation of sunflower oil + *L. plantarum*-R11 with the basal diet; ^2^ SEM: standard error of the mean (*n* = 6 goats per group); ^A,B,C^: mean values within a row with different superscript letters differ significantly (*p* < 0.05).

**Table 7:** The fatty acid composition (g/100g of total FA) of LD muscle of crossbred (Boer × Saanen) goats fed different experimental diets.

Items	Dietary Treatments ^1^					
	Co	T_SFO_	T_LP_	T_SFO+LP_	SEM ^2^	*p*-Value
C14:0, myristic	2.24	2.15	1.53	2.42	0.185	0.151
C16:0, palmitic	21.63	21.41	20.53	20.84	0.632	0.635
C18:0, stearic	14.23	15.22	16.76	17.36	0.873	0.133
C20:0. Arachidic	0.06	0.16	0.10	0.09	0.028	0.387
SFA	38.16	38.94	38.92	40.71	0.690	0.051
C14:1, myristoleic	0.17	0.18	0.08	0.15	0.020	0.118
C16:1, palmitoleic	2.08	2.08	1.53	1.98	0.208	0.342
C18:1 n9t, eladic	1.47	1.91	1.46	1.82	0.295	0.790
C18:1 n9c, oleic	49.61	48.24	48.79	46.07	1.442	0.450
C24:1, nervonic	0.08	0.08	0.10	0.07	0.009	0.109
MUFA	53.41	52.43	51.96	50.09	1.240	0.393
C18:2 n6t, linoelaidic	0.21 ^A^	0.23 ^A^	0.18 ^B^	0.22 ^A^	0.007	0.003
C18:2 n6c, linoleic	5.05	5.08	4.75	5.37	0.700	0.965
C18:3 n6, gamma-linolenic	0.04	0.04	0.05	0.06	0.009	0.419
C18:3 n3, α-linolenic	0.41	0.35	0.36	0.41	0.034	0.691
C20:2, eicosadienoic	0.06	0.07	0.06	0.08	0.010	0.538
C20:3 n6, dihomo-gamma-linolenic	0.21	0.20	0.26	0.18	0.033	0.583
C20:4 n6, arachidonic	1.76	1.92	2.37	2.09	0.307	0.487
C20:5 n3, eicosapentaenoic	0.69	0.73	1.10	0.81	0.201	0.097
Total CLA	0.014 ^B^	0.015 ^B^	0.025 ^A^	0.024 ^A^	0.002	0.028
PUFA	8.43 ^C^	8.62 ^B^	9.13 ^A^	9.22 ^A^	0.021	0.001
n3	1.10	1.08	1.46	1.22	0.177	0.165
n6	7.27	7.47	7.61	7.92	1.021	0.998
PUFA/SFA	0.21	0.22	0.23	0.23	0.030	0.906
ID16	8.77	8.85	6.94	8.68	0.668	0.308
ID18	78.28	76.70	74.98	73.39	2.22	0.194

^1^ C_O_: basal diet; T_SFO_: oral supplementation of sunflower oil + basal diet; T_LP_: oral supplementation of *L. plantarum*-R11 + basal diet; T_SFO+LP_: oral supplementation of sunflower oil + *L. plantarum*-R11 with the basal diet; ^2^ SEM standard error of mean (*n* = 6 goats per treatment); ^A,B,C^: mean values within a row with different superscript letters differ significantly (*p* < 0.05).

**Table 8:** Effects of dietary treatments on serum biochemical indices of crossbred (Boer × Saanen) goats.

Parameter ^1^		Dietary Treatments ^2^				*p*–Value			
	Time	C_O_	T_SFO_	T_LP_	T_SFO+LP_	SEM ^3^	TRT ^4^	Time	TRT × Time
Glucose (mg/dL)	D1	65.0	67.7	63.0	60.7	3.29	ns	ns	ns
	D90	65.0	61.13	61.7	61.7	2.02			
TG (mg/dL)	D1	33.3 ^A^	29.7 ^B^	32.3 ^A^	28.7 ^B^	2.56	*	ns	***
	D90	32.7 ^B^	29.0 ^B^	43.7 ^A^	32.0 ^B^	4.42			
TC (mg/dL)	D1	52.3 ^A^	40.7 ^C^	45.7 ^B^	43.7 ^B^	4.28	***	***	*
	D90	62.3 ^A^	50.3 ^C^	59.0 ^B^	57.3 ^B^	6.22			
HDL (mg/dL)	D1	30.5 ^A^	25.0 ^C^	27.6 ^B^	27.2 ^B^	2.75	***	***	***
	D90	35.7 ^A^	29.0 ^C^	33.5 ^B^	36.4 ^A^	4.27			
LDL (mg/dL)	D1	6.53 ^A^	2.23 ^C^	5.27 ^B^	3.80 ^C^	1.68	***	***	***
	D90	10.67 ^A^	6.50 ^B^	9.73 ^A^	4.93 ^C^	2.25			
ALT (U/L)	D1	102.7 ^B^	110.0 ^B^	125.7 ^A^	108.0 ^B^	23.28	***	***	***
	D90	80.3 ^B^	92.7 ^B^	90.7 ^B^	86.0 ^B^	9.68			
AST (U/L)	D1	25.0 ^B^	24.7 ^B^	29.3 ^A^	24.0 ^B^	3.15	***	***	***
	D90	21.0 ^B^	21.3 ^B^	17.0 ^C^	20.0 ^B^	2.51			

^1^ TG, Triglyceride; TC, total cholesterol, HDL, high density lipoprotein; LDL, low density lipoprotein; ALT, alanine transaminase; AST, aspartate transaminase; ^2^ C_O_: basal diet; T_SFO_: oral supplementation of sunflower oil + basal diet; T_LP_: oral supplementation of *L. plantarum*-R11 + basal diet; T_SFO+LP_: oral supplementation of sunflower oil + *L. plantarum*-R11 with the basal diet; ^3^ SEM: standard error of mean (*n* = 6 goats per treatment); ^4^ TRT: treatments; ns: not significant (*p* > 0.05); * *p* < 0.05; *** *p* < 0.001; ^A,B,C^: mean values within a row with different superscript letters differ significantly (*p* < 0.05).

## Data Availability

Supporting data for the findings reported in this study are available upon request from the corresponding author.

## Supplementary Materials

The following supporting information can be downloaded at: https://www.mdpi.com/article/10.3390/ani16101540/s1, Table S1: Estimated daily dry matter intake (DMI), crude protein (CP), metabolizable energy (ME) intake, and contribution of sunflower oil (SFO) in goats receiving different dietary treatments.
