# Supplementary material for: Dietary Supplementation of Sunflower Oil and Lactiplantibacillus plantarum-R11 Improves Meat Quality and Fatty Acid Composition in Crossbred (Boer × Saanen) Goats
Source: Animals (Basel). 2026 May 18;16(10):1540. doi: 10.3390/ani16101540 (PMC13203947; doi:10.3390/ani16101540)
Supplement: Supplementary file 1 [file animals-16-01540-s001.zip › animals-4235949-supplementary.pdf]

Supplementary Table S1. Estimated daily dry matter intake (DMI), crude protein (CP), metabolizable energy (ME) intake, and contribution of sunflower oil (SFO) in goats receiving different dietary treatments

Items	C_O	T_{SFO}	T_{LP}	T_{SFO+LP}	SEM	p-value
DMI(g/day)	880.23^{A}	768.08^{B}	702.92^{C}	745.85^{B}	24.59	0.020
CP intake (g/day)	91.55	79.88	73.10	77.57	-	-
ME intake (MJ/day)	9.45	8.60	8.05	8.55	-	-
SFO intake (g/day)	0	4.60-9.20	0	4.60-9.20	-	-
SFO energy (MJ/day)	0	0.17-0.34	0	0.17-034	-	-
SFO contribution (% total ME)	0	2.0-3.9	0	2.0-3.9	-	-

C_O: basal diet; T_{SFO}: oral supplementation of sunflower oil + basal diet; T_{LP}: oral supplementation of *L. plantarum*-R11 + basal diet; T_{SFO+LP}: oral supplementation of sunflower oil + *L. plantarum*-R11 with the basal diet; SEM: standard error of the mean (n = 7 goats per treatment); ^{A,B,C} Mean values within a row with different superscript letters differ significantly ($p < 0.05$ or $p < 0.01$).

Calculation notes:

- Crude protein (CP) intake was estimated using the average CP content of the basal diet (~10.4% DM), derived from the weighted contribution of whole corn plant (8.6% CP) and concentrate (13.0% CP).
- Metabolizable energy (ME) was estimated to have an average value of **10.7 MJ/kg DM** for the basal diet, consistent with typical growing goat diets.
- Sunflower oil (SFO) was assumed to provide approximately **37 MJ/kg DM**, and 1 mL ≈ 0.92 g.
- SFO supplementation ranged from **5–10 mL/day**, equivalent to ~4.6–9.2 g/day.
